# Simultaneous enhancement of sweet corn yield and lodging resistance via silicon application with low-phosphorus management under dense planting in tropical China

**DOI:** 10.3389/fpls.2026.1919054

**Published:** 2026-10-20

**Authors:** Qinggan Liang, Hongrong Chen, Jianqiang Wang, Qingdan Wu, Bangliang Deng, Yuanxia Qin, Hao Wen, Xianqing Zeng, Jiantao Wu, Hailong Chang

**Affiliations:** 1 Hainan Institute of Industrial Technology, Guangdong Academy of Science, Sanya, China; 2 Institute of Nanfan & Seed Industry, Guangdong Academy of Sciences, Guangzhou, China; 3 School of Breeding and Multiplication (Sanya Institute of Breeding and Multiplication), Hainan University, Sanya, China

**Keywords:** lodging resistance, phosphorus, silicon, sweet corn, yield

## Abstract

Despite the recognized benefits of silicon (Si) fertilizer in crop production, the agronomic mechanisms underlying Si-mediated improvements in lodging resistance and yield stability under low-phosphorus (P) management in sweet corn remain poorly understood. To address this, a three-season field experiment was conducted using a two-factor split-plot design with two Si levels (Si0: 0 kg ha^-^¹ SiO_2_; Si1: 50 kg ha^-^¹ SiO_2_) as main plots and five P levels (P0-P4: 0–125 kg ha^-^¹ P_2_O_5_) as sub-plots, at a planting density of 60,000 plants ha^-^¹ (33% above local high-yield practice). Our results demonstrated that Si application combined with low-P management (P1: 50 kg ha^-^¹ P_2_O_5_) simultaneously enhanced lodging resistance and yield. This synergistic effect was attributed to four interrelated mechanisms: (1) elevated soil enzyme activities (phosphatase, urease, sucrase, and peroxidase), which might facilitate nutrient and sucrose metabolism; (2) strengthened root-soil anchorage via stimulated brace root formation, with root traits exerting the largest negative direct effect on lodging rate (path coefficient = -0.782, *P*< 0.001); (3) reinforced basal stem node development through enhanced accumulation of structural carbohydrates (lignin, cellulose, and hemicellulose); and (4) optimized canopy architecture, characterized by favorable leaf angles and a greater proportion of leaf area distributed to the middle canopy layer, which was negatively correlated with lodging rate (r = -0.54, *P*< 0.001) and positively correlated with yield (r = 0.39, *P*< 0.01). Collectively, these findings indicate that Si fertilization under low-P regimes offers a promising strategy for improving both stability and productivity in sweet corn production systems.

## 1 Introduction

Sweet corn (*Zea mays* L.) is a major economic crop in tropical China, and the cultivated area of this belt zone is nearly 0.2 million ha, which accounted for 50% of the total national production in 2023 (Liang et al., 2026). Lodging, which can be attributed to extreme weather conditions, such as storms (typhoon) and high temperatures, particularly during the summer growth season (Zhang et al., 2024). Although increasing planting densitycan maximize the yield potential of sweet corn (Liang et al., 2025; Ye et al., 2023), it also aggravates lodging risk by reducing stem strength and root anchorage (Lei et al., 2025). Lodging stress negatively affects the quality of crops by reducing grain number per unit area, grain number, grain weight, etc., and then deteriorates commercial characteristics (Gai et al., 2025; Wu et al., 2021). Hence, improving plant lodging resistance is important in sweet corn production in tropical China.

Optimal fertilizer management can reduce lodging events and increase kernel formation by alleviating shading stress under dense planting. Si fertilization enhances plant growth and yield by mitigating the negative effects of biotic and abiotic stresses in various crops, including rice (Ijaz et al., 2023; Zargar et al., 2019; Kumar et al., 2024), tomato (Wang et al., 2025), pea (Song et al., 2026), rapeseed (Moghadam et al., 2022), sugarcane (Teixeira et al., 2022; Misra et al., 2023), and wheat (Cheraghi et al., 2023). Si fertilizer improves rice plant lodging resistance and yield by optimizing photosynthetic capacity and promoting the development of roots and shoots (Ao et al., 2025). However, only a few studies have tested whether Si fertilizer has the same effect in sweet corn as in rice, particularly in tropical China. Phosphorus (P) is a macro-nutrient, whose deficiency reduces crop yield significantly (Dhillon et al., 2017). The global demand for chemical P is increasing by 2.5–3% every year (Zou et al., 2022); however, most of the applied P is unavailable for plant use because the P applied to the soil becomes fixed (Zou et al., 2022). In the case of rice plants, Si fretilizer increases the dissolution of iron phosphates and releases P into the soil solution in paddy soil (Kumar et al., 2019). Therefore, we speculate that the application of Si fertilizer would improve sweet corn lodging resistance and yield under low P as compare to local practice.

Plant height, ear height, leaf angle, and leaf area distribution strongly influence the canopy structure. Zhao et al. (2022) indicated that optimal plant height and ear height ratio (0.36–0.39) can improve yield and lodging resistance after the application of plant growth retardants. Higher ear height coefficient indicates that the stem subjected to larger external forces, additionally, accompaning with the slender stem, the group plants is susceptible to lodging. However, although a lower ear height ratio decreases lodging risk, it also decreases yield due to the low photosynthetic productivity at middle-low canopy leaves. Leaf area (LA) distribution strongly influences canopy photosynthetic assimilation and lodging resistance of cereal crops (Zhao et al., 2025).While the spatial distribution of leaf angle and area throughout the canopy plays a major role in lodging resistance and yield improvement, how these traits dynamically respond to Si and P application under dense planting conditions is still unknown.

Root-shoot interaction strongly influences plant lodging and yield. Zhang et al. (2021) reported that root anchorage is positively correlated with stem bending strength in maize, and thus they speculated that root-shoot interaction probably regulates plant lodging, while the opposite conclusion was discussed by Xue et al. (2020). The architecture of the root system, such as root angle, root number, and root dry weight, is positively correlated with plant lodging (Bian et al., 2016). The root system of maize consists of brace roots and crown roots (Zhan et al., 2019). Brace roots support plant upright, whereas crown roots absorb water and nutrients (Hostetler et al., 2021). Hence, balancing the differentiation and development of the root system can effectively improve lodging resistance and yield output. Stem node development, including stem diameter, stem length, and stem node strength, strongly affects plant lodging (Zhang et al., 2025). A high content of structural carbohydrates (lignin, cellulose, and hemicellulose) in the stem node is positively correlated with the plant lodging resistance (Zhang et al., 2020). However, most research on Si fertilization has concentrated on stem traits (mechanical strength and lignin metabolism), whereas root differentiation and structural carbohydrate metabolism in roots have captured little attention.

In this study, we hypothesized that Si application can improve the yield and lodging resistance of sweet corn by coordinating the development of roots and shoots, structural carbohydrate metabolism, and canopy architecture under low P levels and dense planting density in different environmental condition in tropical China. The objectives of this study were as follows: (1) uncover the agronomic response underlying silicon-mediated improvements in sweet corn yield and lodging resistance under low P and high planting density; (2) provide a theoretical foundation for Si fertilization strategies; and (3) guide the development of ideotypic plant architectures for sweet corn breeding.

## 2 Material and methods

### 2.1 Experimental design

A three-round field experiment was conducted at the research base of the Institute of Nanfan & Seed Industry, Guangdong Academy of Sciences, in Yazhou District, Sanya, Hainan, China (18°21′30″N, 109°9′54″E). The three rounds (Exp. 1-3) were carried out during the following periods: Jun 1-Aug 25, 2025; Nov 5, 2025–Feb 7, 2026; and Dec 7, 2025–Mar 5, 2026, respectively. The soil chemical properties at the 0–20 cm depth were as follows: pH 6.73, organic matter 1.22%, alkaline-hydrolyzable nitrogen 62.44 mg kg^-1^, available phosphorus 29.01 mg kg^-1^, available potassium 48.66 mg kg^-1^, and water-extractable silicon 21.56 mg kg^-1^. The climatic conditions during the three growth seasons are presented in **Figure 1**. A two-factor split-plot design was adopted in this study. Two Si levels (Si0: 0 kg ha^-1^ Si; Si1: 50 kg ha^-1^ Si) were used in the main plot; five P application levels, where P3 was local practices (P0: 0 kg ha^-1^ P_2_O_5_; P1: 50 kg ha^-1^ P_2_O_5_; P2: 75 kg ha^-1^ P_2_O_5_; P3: 100 kg ha^-1^ P_2_O_5_; P4: 125 kg ha^-1^ P_2_O_5_) were used in the subplots, each plot was 30 m^2^, and each treatment was performed in triplicate. The sweet corn variety was Jinbaitian15 (JBT15; purchased from Jinmama Agricultural Technology Co., Ltd., Qingdao, China) which cultivated widely in tropical China. The planting density in local high-yield fields is 40,000–50,000 plants ha^-1^. In this study, the planting density was set at 60,000 plants ha^-1^ (increase 33.33%). Moreover, 200 kg ha^-1^ nitrogen (N) and 100 kg ha^-1^ potassium (K), along with the above-mentioned fertilizers were applied before sowing. The fertilizer resources used were: urea containing 46% N (SINOPEC, Co., Ltd.), calcium superphosphate containing 16% P_2_O_5_ (SDIC Xinjiang Lop Nur Potassium Salt Co., Ltd.), and potassium sulfate with 52% K_2_O (Guangdong Zhanhua Group Co., Ltd.); water-soluble silicon fertilizer with 50% SiO_2_ (SINOPEC, Co., Ltd., **Supplementary Figure S1**). P_2_O_5_; SDIC Xinjiang Lop Nur Potassium Salt Co., Ltd.), and potassium sulfate (52% K_2_O; Guangdong Zhanhua Group Co., Ltd.). We germinated the seeds in 105-well plug trays filled with nutrient-rich substrate and transplanted the seedlings to the field at the three-leaf stage. Field management followed local high-yielding field practices.

**Figure 1:**
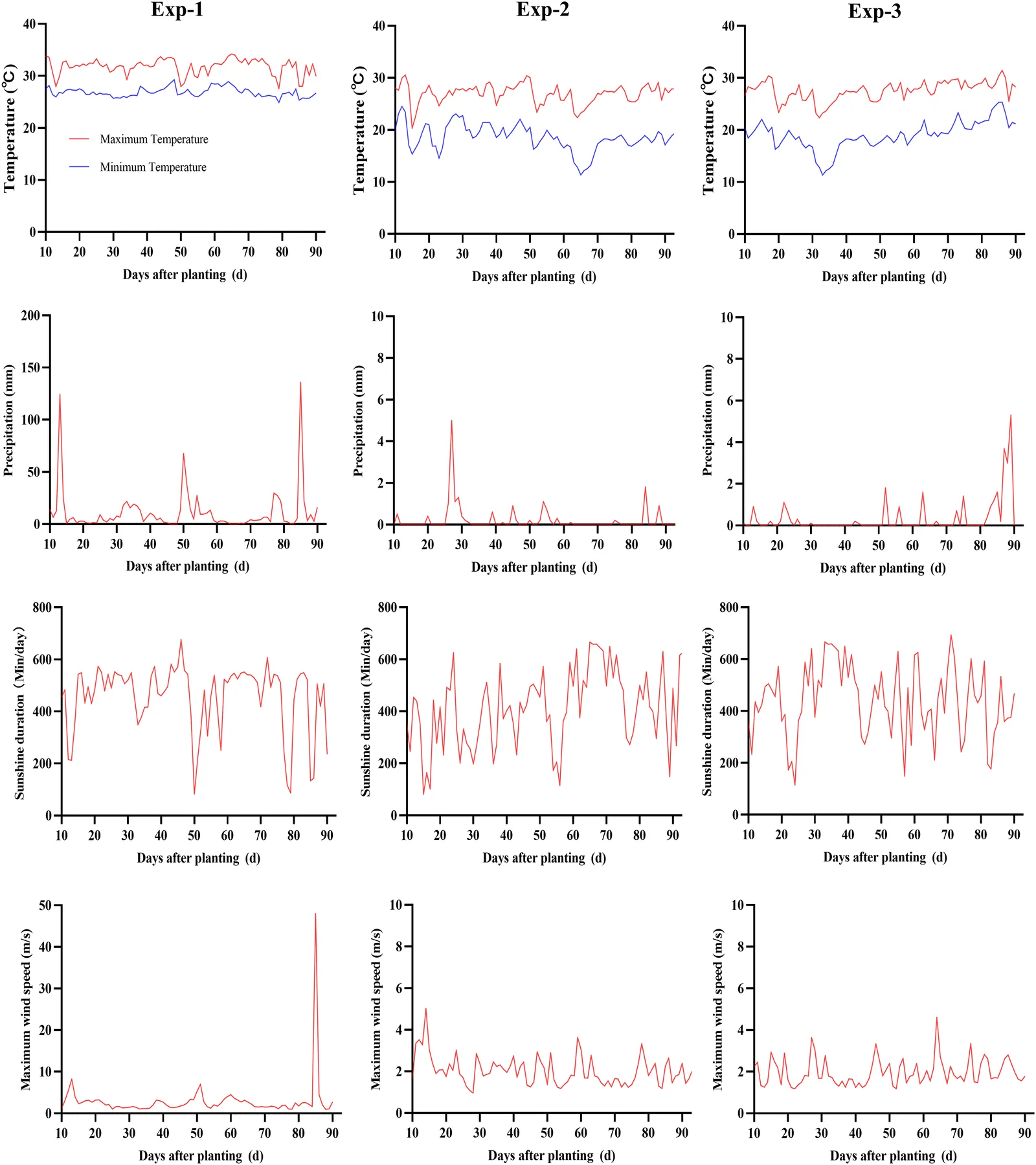
Climate data during three growth seasons.

### 2.2 Measurement procedures

Root architecture, stem node traits, and structural carbohydrate contents in both stems and roots were assessed at the silking stage.The plant height, ear height, leaf area, and soil enzyme activity were measured in the filling stage. The yield and yield components were investigate during the harvest period (21 days after pollination period). The lodging event occurred during the filling stage of Exp-2. Although natural lodging did not occur in Exp-3, we tested the lodging potential artificially.We used an unmanned aerial vehicle (DJI drone, Shenzhen Dajiang Innovation Technology Co., Ltd.) to simulate strong winds and flooding irrigation to simulate soil moisture (100% when heavy rain as in Exp-2) to recreate the scenario in Exp-2 as much as possible during the filling stage (the same growth duration) in Exp-3. The flight parameters were as follows: wind speed, 15 m/s; treatment duration, 5 min per plot; drone height, 50 cm above the tassel tip; and flight pattern, parallel to the row direction (see **Supplementary Figure S2** for details). All plants were lodged, and no harvest occurred in Exp. 1 due to the super typhoon “Kajiki” (seriously affected Sanya City on 24 Aug, 2025; 50 m/s wind speed, **Supplementary Figure S3**). The plant tilted over 30°from vertical is defined as lodged plant through the field experiments.

#### 2.2.1 Root traits

According to our previous study (Liang et al., 2026; Liang et al., 2025), a metal frame (30 cm × 20 cm × 30 cm; length×width×height) was used to collect the whole root system, with soil bulk excavated from the top 0–30 cm soil layer (over 90% roots were distribute within the layer). Then, all roots with soil bulk were collected in a bucket. The root system was soaked in clean running water to remove the soil and other impurities. The primary root system and residual fine roots were collected and air-dried for 30 min. The root angles (narrow and wide sides) were measured by a protractor, brace root number (BRN), brace root whorl number (BRWN), and crown root number (CRN) were recorded. The brace root number ratio (BRNR) and crown root number ratio (CRNR) were also calculated as followed Equations 1, 2, respectively.

(1) BRNR = BRN/TRN

(2) CRNR = CRN/TRN

Where, TRN indicates the total root number.

Each root sample was placed in an envelope, dried at 80 °C for 48 h until a constant dry weight was achieved, and then used for structural carbohydrate analysis.

#### 2.2.2 Stem nodes characteristics

The diameter and length of the first, third, fifth, and seventh inter-nodes were measured using vernier calipers and a tape ruler, respectively. The bending strength, compressive strength, and puncture strength were measured using a digital force tester (YYD-1, Zhejiang Top Instrument, Hangzhou, Zhejiang, China).

(3) The flatness of the stem node=a/b 

(Wu et al., 2020)

(4) The cross−sectional area of the stem node=π×a×b/4

(Wu et al., 2020)

Where, “a” and “b” indicate the outer diameters of the minor and major axes in an oval cross-section, respectively.

The length and width of leaves in different canopy layers, plant height, ear height, and ear height coefficient (EHC) were measured and calculated as follows:

(5) LA = length×width×0.75

(Li et al., 2022)

(6) EHC= ear height/plant height

(Bian et al., 2024)

After the measurement, the third stem node was recollected in an envelope and heated at 80 °C for 48 h till it reached a constant dry weight. The dry sample was used for determining the structural carbohydrate content.

#### 2.2.3 *S*tructural carbohydrate content assay

The dried segments of root and stem nodes were subsequently pulverized using a 0.5 mm sieve. The cellulose content (CC), hemicellulose content (HCC) and the lignin content (LC) were determined by Wuhan ProNets Testing Technology Co., Ltd.

#### 2.2.4 Lodging rate

During the harvest period, the number of lodged plants (LPN) and total plants (TPN) were accounted from each plot, and the lodging rate was calculated as follows:

(7) LR (%)=(LPN/TPN)×100%

(Bian et al., 2024)

#### 2.2.5 Rhizosphere soil enzyme activities

Rhizosphere soil was collected by shaking roots to remove soil adhering to the root surface, passed through a 2-mm sieve, and stored at –80 °C for enzyme analysis. The activities of rhizosphere soil enzymes were measured using modified techniques. SUR activity was measured by indophenol colorimetry with urea as the substrate, following the method described by Kandeler and Gerber (1988). Ammonium released over 1 h was analyzed colorimetrically at 578 nm. SPH activity was measured by disodium phenyl phosphate colorimetry following the method described by Niu et al. (2015). The amount of phenol released over 1 h was analyzed colorimetrically at 660 nm. The peroxidase (POD) activity was measured by the p-nitrophenol colorimetry method. The activities of cellulase (CL) and sucrase (SU) were determined using detection kits purchased from Nanjing Jiancheng Biotechnology Research Institute Co., Ltd. (Nanjing, Jiangsu Province, China). Three biological replicates were established for each treatment.

#### 2.2.6 Yield and yield components

For yield determination, ears harvested from the central three rows of each plot were counted and weighed for fresh weight. The commercial characteristics of ears, including corn ear length, ear diameter, cob length (ear without husk), cob diameter (ear without husk), row number per ear, grain number, grain number per row, and barren tip length, were subsequently measured. Coefficients of variation (Cv) of these commercial traits were used to represent the yield stability as follows:

(8) Cv(%)=(standard deviation(SD)/mean)

(Han et al., 2020)

A smaller CV indicates that the consistency of the data is better.

### 2.3 Statistical analysis

The effect of P, Si, seasons (S) and their interaction (P×Si×S) on all variables was determined by conducting three-way analysis of variance (ANOVA) at the significance levels of *P*< 0.05, *P*< 0.01, and *P*< 0.001. The differences between means were determined by conducting Duncan’s multiple range test at the level of significance of *P<* 0.05. Pearson product-moment correlation coefficients (r) and significance of the associations (*P<* 0.05) were calculated between lodging and root-shoot traits by SPSS 19.0. (IBM, SPSS, Statistic 19, USA). Structural equation modeling (SEM) was performed in SPSS AMOS 24.0 (SPSS Inc., Chicago, IL, USA). The data were visualized and presented using GraphPad Prism 8.1.4 for Windows and expressed as the mean and standard errors (Mean ± SE).

## 3 Results

### 3.1 Root differentiation and characteristics

Phosphorus (P) and silicon (Si) fertilization significantly influenced root differentiation and development in sweet corn. Across the three growing seasons, both BRN and CRN were highest under P1Si1 (**Figures 2A–F**; *P*< 0.05). BRN and CRN peaked at P1 and then declined with increasing P supply (**Figures 2A–F**; *P*< 0.05). On average, Si1 increased BRN by 43.33% (15.07 vs. 21.60), 24.33% (16.07 vs. 19.98), and 19.39% (18.93 vs. 22.60) across the three seasons; the corresponding increases for CRN were 26.93% (22.87 vs. 29.03), 13.24% (17.60 vs. 19.93), and 21.27% (19.46 vs. 23.60) (**Figures 2A–F**; *P*< 0.05). In Exp-1 and Exp-3, plants in Si1 plots had significantly higher BRNR than those in Si0 plots, but no significant difference was observed in Exp-2 (**Figures 2G–I**; *P*< 0.05). Conversely, the opposite pattern was found for CRNR in Exp-1 and Exp-3 (**Figures 2J–L**; *P*< 0.05).

**Figure 2:**
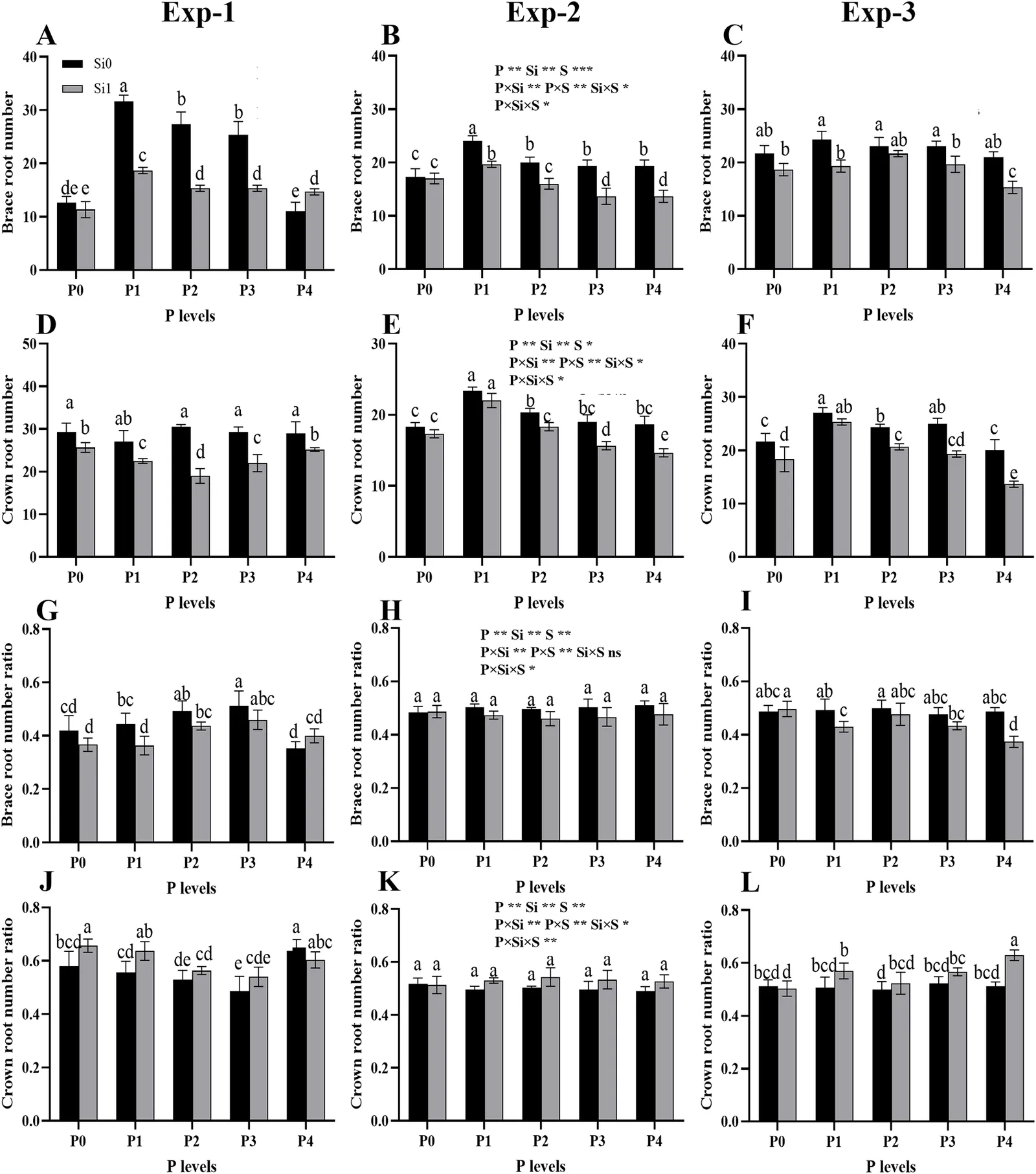
The effect of applying P and Si on the development of sweet corn roots under dense planting conditions during three growth seasons is illustrated. Brace root number **(A-C)**; crown root number **(D-F)**; brace root number ratio **(G-I)**; crown root number ratio **(J-L)**. Vertical bars represent SE values. Lowercase letters in the figure indicate significant differences among different treatments determined by Duncan’s test (*P*< 0.05). Three-way Anova was conducted to evaluate effect of P, Si, Season and their interaction (P×Si×S) on variables at the significance levels of *P*< 0.05, *P*< 0.01, *P*< 0.001, and no significant difference (P > 0.05), represented by “*”, “**”,”***”, and “ns”, respectively.

P and Si fertilization significantly affected root angle across the three growing seasons (**Figure 3**). For the wide-side angle, it decreased with increasing P and Si supply, with the largest values consistently recorded in P0Si1 across all seasons (**Figures 3A–C**; *P*< 0.05); these values were comparable to those in P1Si1 during Exp-1 and Exp-3 (*P*< 0.05). On average, Si1 increased the wide-side angle by 17.04%, 12.51%, and 6.65% across the three seasons (**Figures 3A–C**; *P*< 0.05). For the narrow-side angle, the maximum occurred in P1Si1, which was similar to P2Si1 in Exp-1 and to P0Si1 in Exp-2 (**Figures 3D–F**; P<0.05P<0.05). Si1 increased the narrow-side angle by 19.06%, 10.39%, and 4.22% on average across the three seasons (**Figures 3D–F**; *P*< 0.05).

**Figure 3:**
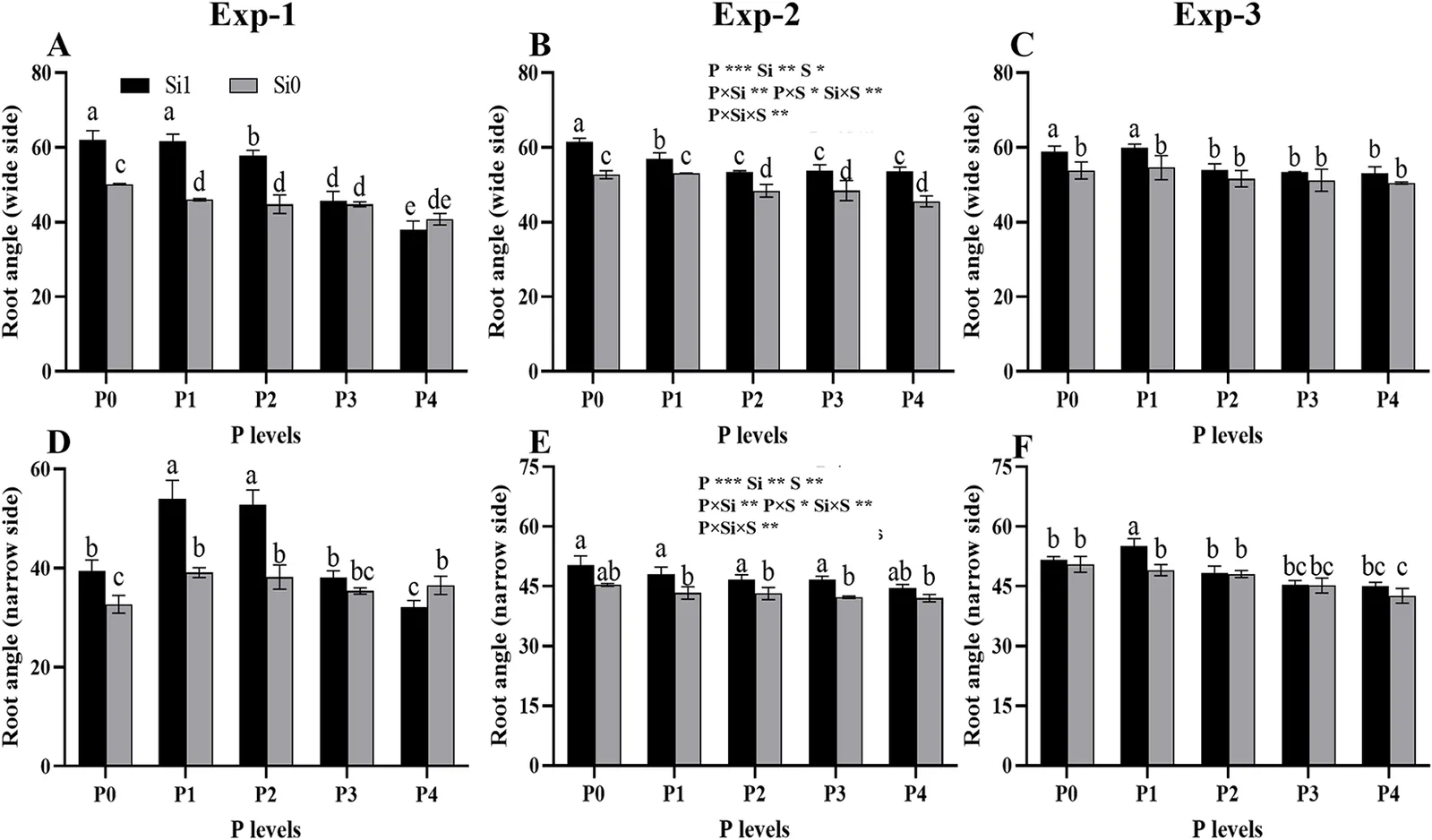
The effect of applying P and Si on the root angle of sweet corn under dense planting conditions during three growth seasons is illustrated. Root angle (wide side) **(A-C)**; root angle (narrow side) **(D-F)**. Vertical bars represent SE values. Lowercase letters in the figure indicate significant differences among different treatments determined by Duncan’s test (*P*< 0.05). Three-way Anova was conducted to evaluate effect of P, Si, Season and their interaction (P×Si×S) on variables at the significance levels of *P*< 0.05, *P*< 0.01, *P*< 0.001, and no significant difference (P > 0.05), represented by “*”, “**”,”***”, and “ns”, respectively.

Maximum BRWN was observed in the P1Si1 plots during the growth seasons corresponding to Exp-2 and Exp-3 (**Figure 4**; *P*< 0.05). The average BRWN in the Si1 plots was 2.2 and 2.26, which was 4.76% and 21.50% higher than those in the Si0 plots in Exp-2 and Exp-3, respectively (**Figure 4**; *P*< 0.05).

**Figure 4:**
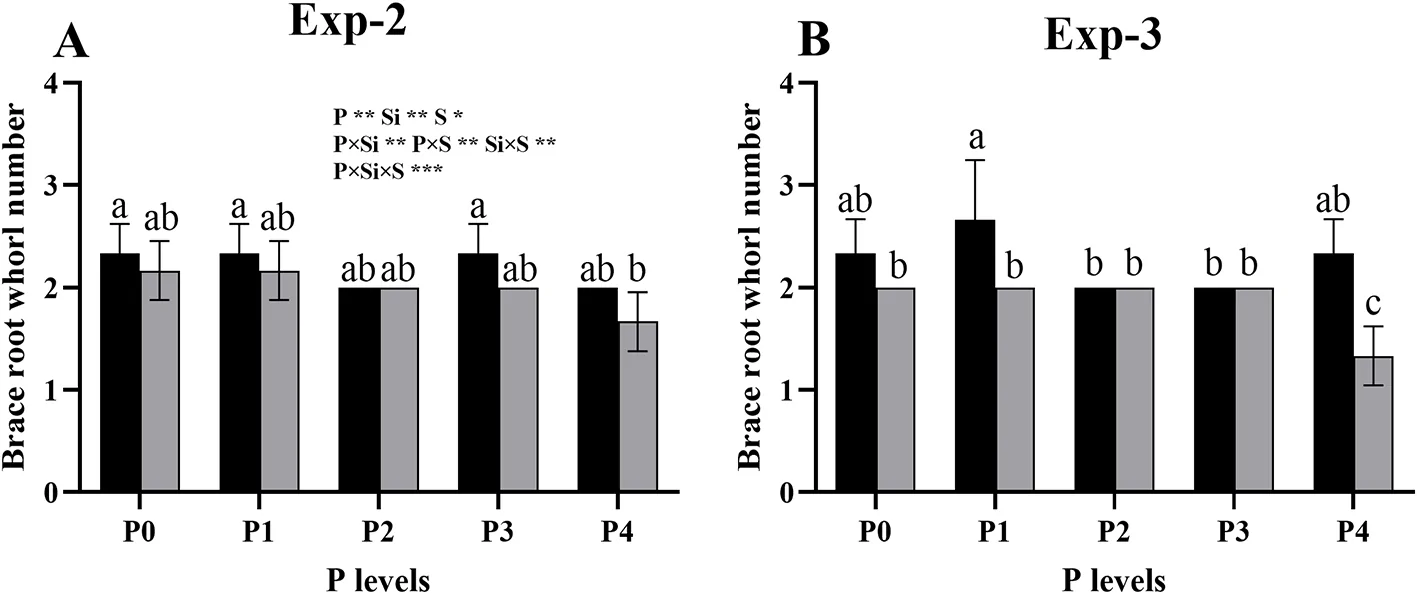
The effect of P and Si application on the brace root whorl number of sweet corn under dense planting conditions during the growth seasons (Exp-2 and Exp-3) is illustrated. Exp-2 **(A)**; Exp-3 **(B)**. Vertical bars represent SE values. Lowercase letters in the figure indicate significant differences among different treatments determined by Duncan’s test (P< 0.05). Three-way Anova was conducted to evaluate effect of P, Si, Season and their interaction (P×Si×S) on variables at the significance levels of P< 0.05, P< 0.01, P< 0.001, and no significant difference (P > 0.05), represented by “*”, “**”,”***”, and “ns”, respectively.

### 3.2 Plant height, ear height and ear height coefficient

The P and Si fertilizers significantly affected plant height, ear height, and EHC across the three growth seasons (**Figure 5**). The plant height of sweet corn varied with the growth seasons (**Figure 5A-C**); during Exp-3, the height of the plants in the Si1 plots was significantly lower (22.53%; 224.24 vs. 274.78) than that of the plants in the Si0 plots when the same level of P was applied (**Figure 5C**; *P*< 0.05). By contrast, the ear height and EHC increased considerably in plants in the Si0 plots when the same level of P was applied. On average, the ear height in the Si0 plots was 2.09% (88.74 vs. 90.6), 6.74% (89 vs. 95), and 20.13% (87.7 vs. 105.36) higher than that in the Si1 plots during the three growth seasons, respectively (**Figure 5D-F**; *P*< 0.05). The increase in EHC in Si0 was 5.87% (0.409 vs. 0.433), 5.80% (0.362 vs. 0.383), and 11.71% (0.35 vs. 0.391), respectively (**Figure 5G-I**; *P*< 0.05).

**Figure 5:**
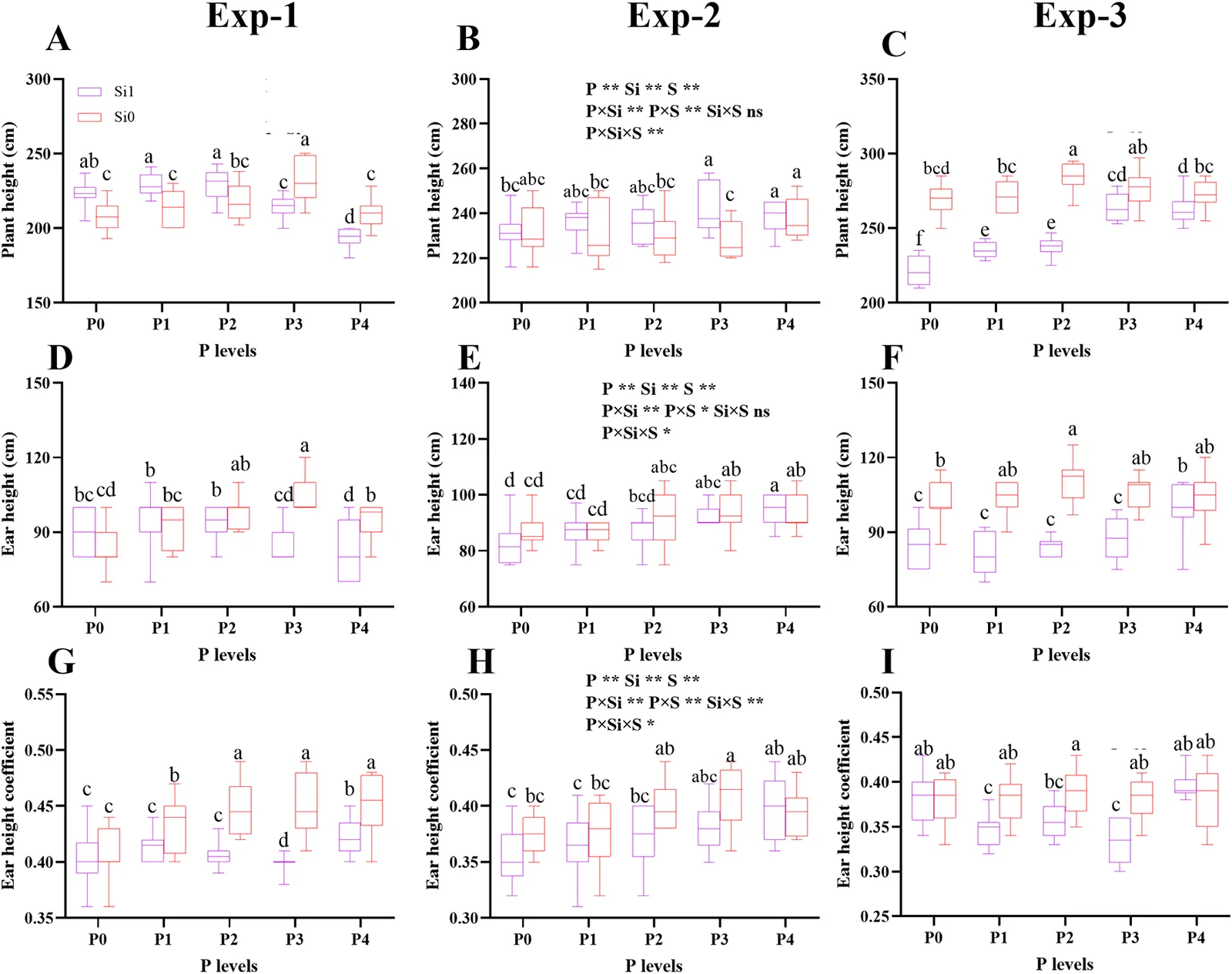
The effect of P and Si application on the plant height, ear height, and ear height coefficient of sweet corn under dense planting conditions during three growth seasons is illustrated. Plant height **(A-C)**; ear height **(D-F)**; ear height coefficient **(G-I)**. Vertical bars represent SE values. Lowercase letters indicate significant differences among different treatments determined by Duncan’s test (*P*< 0.05). Three-way Anova was conducted to evaluate effect of P, Si, Season and their interaction (P×Si×S) on variables at the significance levels of *P*< 0.05, *P*< 0.01, *P*< 0.001, and no significant difference (*P* > 0.05), represented by “*”, “**”,”***”, and “ns”, respectively.

### 3.3 Stem node characteristics and mechanical strength

P and Si fertilization significantly affected stem development (**Figure 6**). Third-internode length increased with increasing P rate, and was significantly greater in Si0 than in Si1 across treatments (**Figure 6A-C**). For third-internode diameter, the maximum occurred in P1Si1, with Si1 significantly exceeding Si0 in Exp-1 and Exp-2, but not in Exp-3 at the same P level (**Figures 6D–F**; *P*< 0.05). Internode flatness showed no significant difference between Si1 and Si0 under the same P application (**Figure 6G-I**). In contrast, the cross-sectional area of the third internode was significantly increased by Si1 compared with Si0 at the same P level (**Figure 6J-L**; *P*< 0.05).

**Figure 6:**
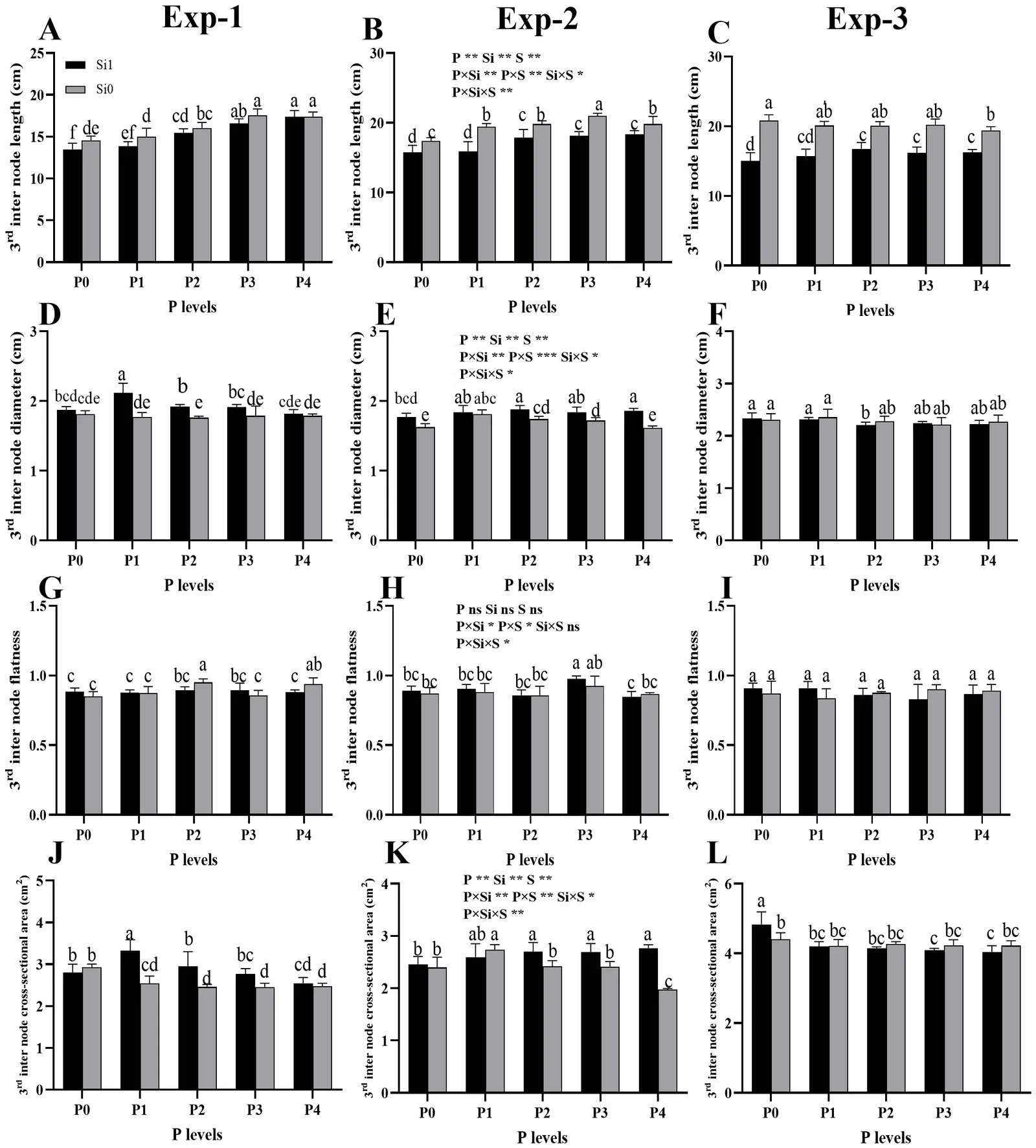
The effect of P and Si application on the development of the third stem node of sweet corn under dense planting conditions during the three growth seasons is illustrated. Third stem node length **(A-C)** third stem node diameter **(D-F)** third stem node flatness **(G-I)** third stem node cross-sectional area **(J-L)**. Vertical bars represent SE values. Lowercase letters indicate significant differences among different treatments determined by Duncan’s test. Three-way Anova was conducted to evaluate effect of P, Si, Season and their interaction (P×Si×S) on variables at the significance levels of *P*< 0.05, *P*< 0.01, *P*< 0.001, and no significant difference (P > 0.05), represented by “*”, “**”,”***”, and “ns”, respectively.

The P and Si fertilizers significantly affected the mechanical strength of the stem nodes during the three growth seasons (**Figure 7**). The bending strength, compressive strength, and puncture strength of the third internode were significantly higher in the Si1 plots than in the Si0 plots, and the plants in the P1Si1 plots presented the maximum value (except for bending strength in Exp-1 and puncture strength in Exp-2) (**Figure 7**; *P*< 0.05).

**Figure 7:**
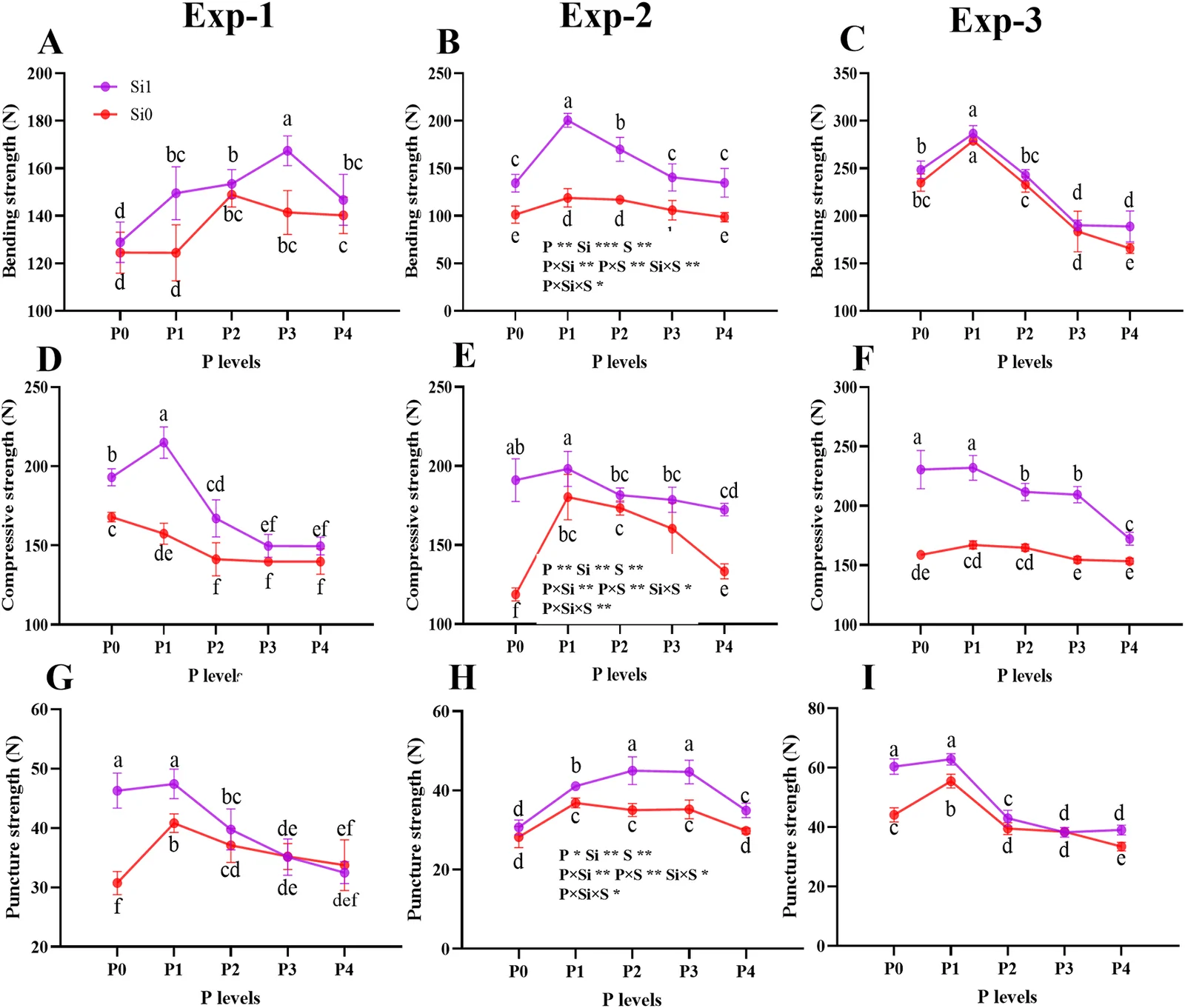
The effect of P and Si application on the third stem node mechanical strength of sweet corn under dense planting conditions during the three growth seasons is illustrated. Bending strength **(A-C)** compressive strength **(D-F)** puncture strength **(G-I)**. Vertical bars represent SE values. Lowercase letters indicate significant differences among different treatments determined by Duncan’s test (*P*< 0.05). Three-way Anova was conducted to evaluate effect of P, Si, Season and their interaction (P×Si×S) on variables at the significance levels of *P*< 0.05, *P*< 0.01, *P*< 0.001, and no significant difference (P > 0.05), represented by “*”, “**”,”***”, and “ns”, respectively.

### 3.4 Leaf area and distribution ratio through canopy layers

P and Si fertilization significantly affected canopy leaf area distribution across the growing seasons (**Figure 8**). For the lower canopy, leaf area was significantly greater in Si0 than in Si1 under the same P level (**Figure 8A**; *P*< 0.05), with average increases of 15.67% (0.30 vs. 0.347) and 22.72% (0.308 vs. 0.378) in Exp-2 and Exp-3, respectively (**Figures 8D, E**; *P*< 0.05). For the middle canopy, leaf area increased with P rate, with the greatest values in P4Si1 (Exp-2) and P3Si1 (Exp-3) (**Figure 8A**; *P*< 0.05); Si1 significantly exceeded Si0 under the same P level, by 5.83% (0.480 vs. 0.508) and 12.14% (0.494 vs. 0.554) on average across the two seasons (**Figures 8D, E**; *P*< 0.05). For the upper canopy, leaf area showed a similar seasonal trend in Exp-2 and Exp-3, peaking in P3Si1 (**Figure 8C**; *P*< 0.05); Si1 increased leaf area by 9.30% (0.172 vs. 0.188) and 8.73% (0.126 vs. 0.137) compared with Si0 on average (**Figures 8D, E**; *P*< 0.05).

**Figure 8:**
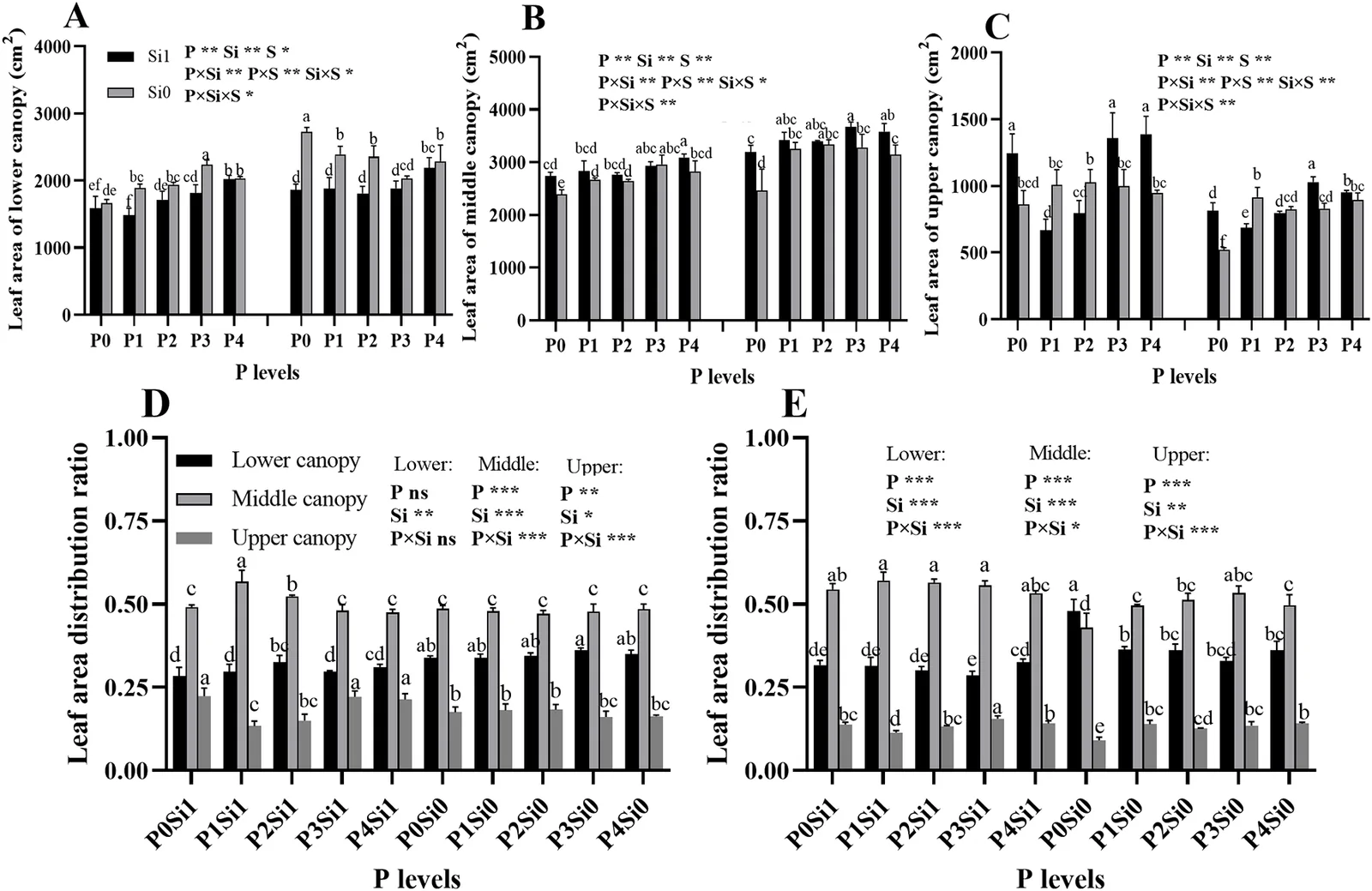
The effect of P and Si application on the leaf area and its distribution in sweet corn through canopy layers under dense planting conditions during two growth seasons (Exp-2 and Exp-3) is illustrated. Leaf area of lower canopy **(A)** leaf area of middle canopy **(B)**; leaf area of upper canopy **(C)**; leaf area distribution ratio in Exp-2; **(D)** leaf area distribution ratio in Exp-3 **(E)**. Vertical bars represent SE values. Lowercase letters indicate significant differences among different treatments determined by Duncan’s test (*P*< 0.05). Three-way Anova was conducted to evaluate effect of P, Si, Season and their interaction (P×Si×S) on variables at the significance levels of *P*< 0.05, *P*< 0.01, *P*< 0.001, and no significant difference, represented by “*”, “**”,”***”, and “ns”, respectively.

### 3.5 Structural carbohydrates content

P and Si fertilization significantly affected structural carbohydrate contents in the third internode and brace roots (**Figure 9**). Lignocellulose (LC) content in both organs was highest in P1 plants and was significantly greater in Si1 than in Si0 under the same P level (**Figures 9A, D**; *P*< 0.05), with average Si1-induced increases of 44.79% (96 vs. 139 mg g^-^¹) in internodes and 12.80% (125 vs. 141 mg g^-^¹) in brace roots (**Figures 9A, D**; *P*< 0.05). Cellulose (CC) content in the third internode was significantly higher in Si1P2 and Si1P3, with a mean increase of 21.65% (314 vs. 382 mg g^-^¹) under Si1 (**Figures 9B, E**; *P*< 0.05). In brace roots, however, CC was highest in P2Si0, and Si1 had only a marginal effect (+1.01%, 297 vs. 300 mg g^-^¹; not significant). Hemicellulose content peaked in P2Si1 in both organs, with Si1 increasing it by 14.67% (109 vs. 125 mg g^-^¹) in internodes and 16.98% (106 vs. 124 mg g^-^¹) in brace roots on average (**Figure 9C-F**; *P*< 0.05).

**Figure 9:**
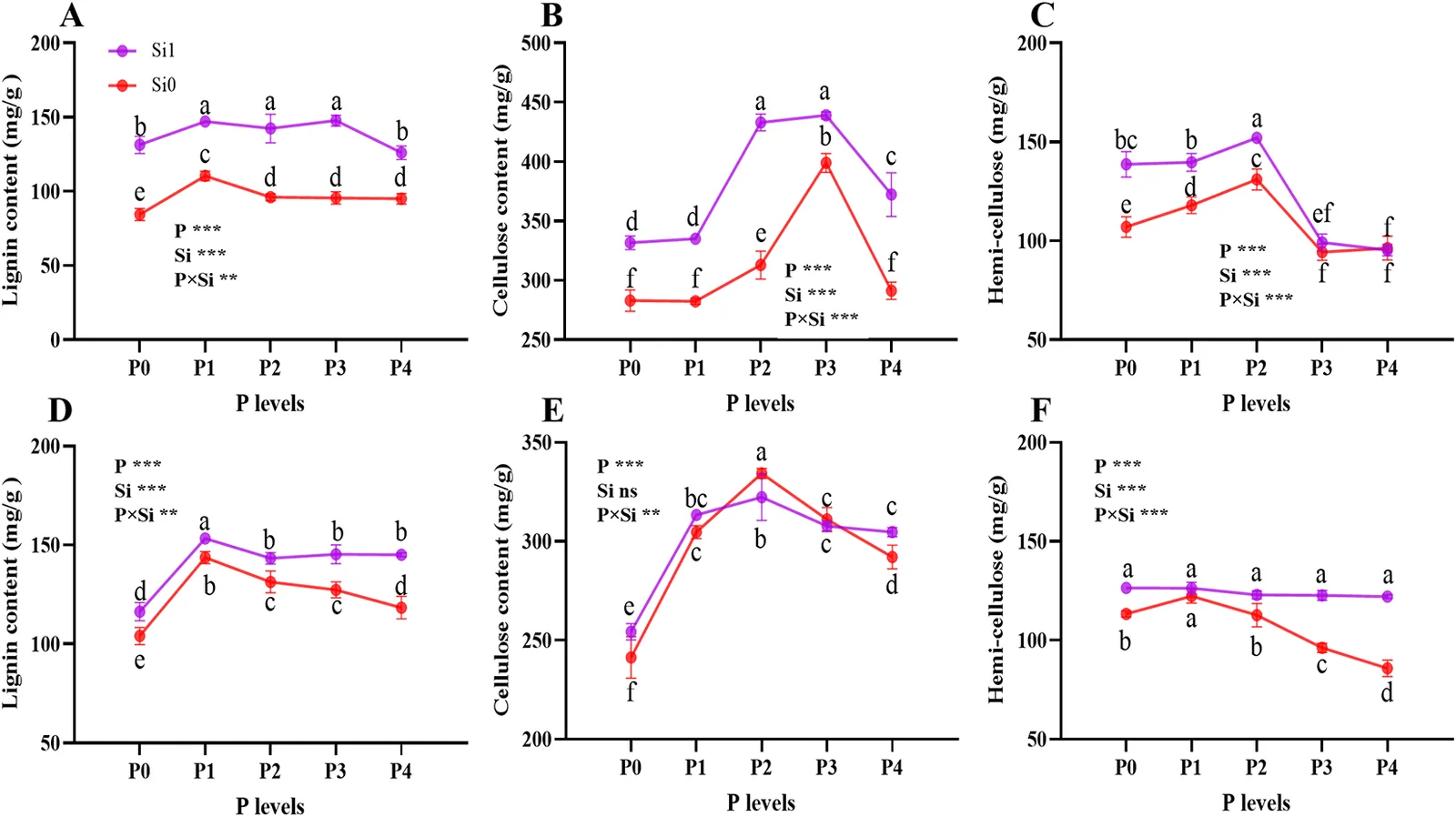
The effect of P and Si application on structural carbohydrates metabolism in the third internode **(A-C)** and brace root **(D-F)** in Exp-2 is illustrated. Lignin content in the third internode **(A)** and brace root **(D)** Cellulose content in the third internode **(B)** and brace root **(E)** hemicellulose content in the third internode **(C)** and brace root **(F)**. Vertical bars represent SE values. Lowercase letters indicate significant differences among different treatments determined by Duncan’s test (*P*< 0.05). Two-way ANOVA was conducted to evaluate the effect of P, Si, and their interaction (P×Si) on variables at the significance levels of *P*< 0.05, *P*< 0.01, *P*< 0.001, and no significant difference (P > 0.05), represented by “*”, “**”,”***”, and “ns”, respectively.

### 3.6 Soil enzyme activity

P and Si fertilization significantly affected soil enzyme activities (**Figure 10**). Acid phosphatase (SPH), urease, sucrase (SU), and peroxidase (POD) activities were significantly increased by Si application (**Figures 10A–C, E**; *P*< 0.05). Phosphatase and urease activities peaked in P*1Si1, whereas SU and POD activities were highest in P2Si1 and P0Si1, respectively (***Figures 10A–C, E***; P*< 0.05). Cellulase (CL) activity, however, showed no significant difference between Si1 and Si0 under the same P level (**Figure 10D**; *P*>0.05). On average, Si1 increased SPH, urease, SU, and POD activities by 18.04% (24 vs. 28.33 U g^-^¹), 5.88% (136 vs. 144 U g^-^¹), 58.14% (8.6 vs. 13.6 U g^-^¹), and 40.91% (0.66 vs. 0.93 U g^-^¹), respectively (**Figures 10A–C, E**; *P*< 0.05), respectively.

**Figure 10:**
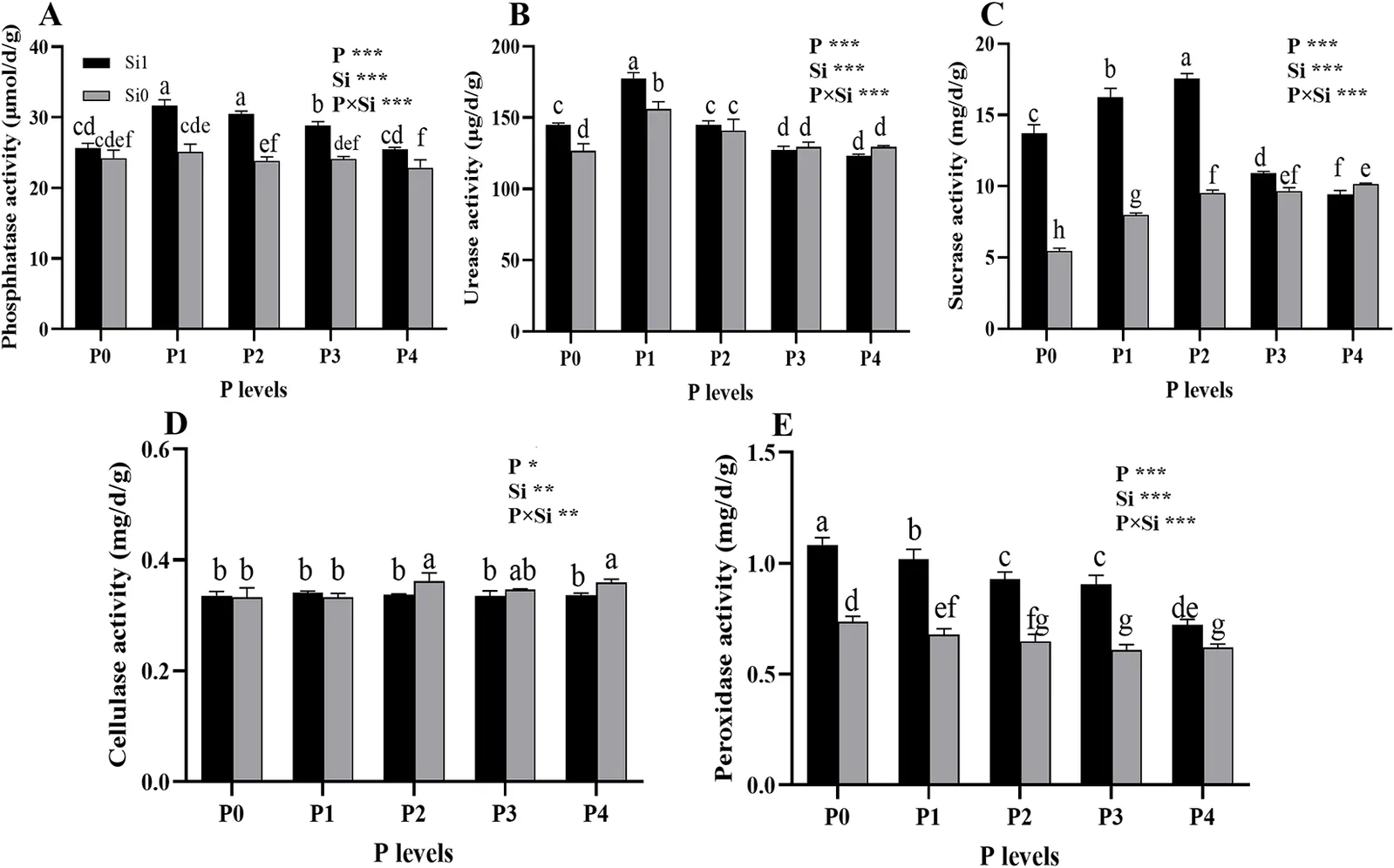
The effect of P and Si application on the activity of soil enzymes in Exp-2 is illustrated. Phosphatase activity **(A)** urease activity **(B)** sucrase activity **(C)** cellulase activity **(D)** peroxidase activity **(E)**. Vertical bars represent SE values. Lowercase letters indicate significant differences among different treatments determined by Duncan’s test (*P*< 0.05). Two-way ANOVA was conducted to evaluate the effect of P, Si, and their interaction (P×Si) on variables at the significance levels of *P*< 0.05, *P*< 0.01, *P*< 0.001, and no significant difference (P > 0.05), represented by “*”, “**”,”***”, and “ns”, respectively.

### 3.7 Lodging rate

The P and Si fertilizers strongly influenced the lodging resistance in sweet corn (**Figure 11**). The lodging rate of Si1 plants was significantly lower than that of Si0 plants (**Figure 11**; *P*< 0.05). The lowest lodging rate was observed in the P1Si1 plants (**Figure 11**; *P*< 0.05). Compared to that in the Si0 plants, the lodging rate in Si1 plants decreased by 35.10% (19.57% vs. 26.44%) and 160.34% (4.69% vs. 12.21%) on average during the growth periods corresponding to Exp-2 and Exp-3, respectively (**Figure 11**; *P*< 0.05).

**Figure 11:**
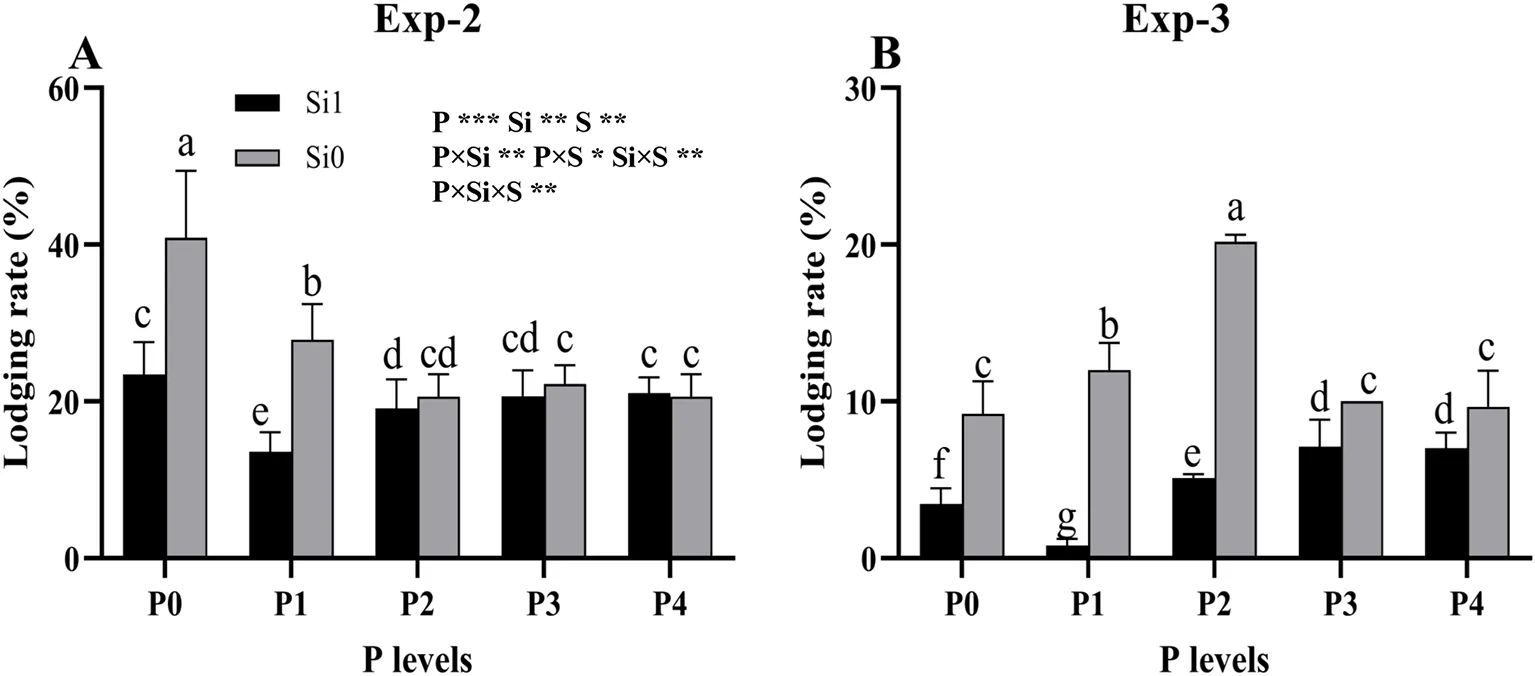
The effect of P and Si application on the lodging of sweet corn during the Exp-2 **(A)** and Exp-3 **(B)** growth periods is illustrated. Vertical bars represent SE values. Lowercase letters indicate significant differences among different treatments determined by Duncan’s test (*P*< 0.05). Three-way Anova was conducted to evaluate effect of P, Si, Season and their interaction (P×Si×S) on variables at the significance levels of *P*< 0.05, *P*< 0.01, *P*< 0.001, and no significant difference (P > 0.05), represented by “*”, “**”,”***”, and “ns”, respectively.

### 3.8 Yield, yield component and commercial traits

P and Si fertilization significantly increased ear yield, row number per ear (RNPE), kernel number per row (KNPR), and grain number per ear (GNPE) in Si1 compared with Si0 at the same P level during Exp-2 and Exp-3 (**Table 1**; *P*< 0.05). In Exp-2, Si1 increased these parameters by 19.03% (22.63 vs. 26.94), 4.74% (15.20 vs. 15.92), 2.23% (35.80 vs. 36.60), and 6.81% (543 vs. 580), respectively. In Exp-3, the corresponding increases were 3.47% (25.92 vs. 26.82), 2.55% (17.24 vs. 17.68), 4.76% (35.28 vs. 36.96), and 10.39% (587 vs. 648) (**Table 1**; *P*< 0.05).

**Table 1:** Effects of nitrogen level (N), planting density (P), and year (Y) on ear yield and yield components.

Growth seasons	Si levels	P levels	Yield (t/ha)	Cv	RNPE	Cv	KNPR	Cv	GNPE	Cv
Exp-2	Si1	P0	26.88 a	0.035	16.80 a	0.058	36.6 ab	0.084	594.8 ab	0.103
		P1	27.06 a	0.017	15.20 ab	0.064	37.8 ab	0.085	570.0 ab	0.098
		P2	27.06 a	0.011	15.20 ab	0.105	37.8 ab	0.155	571.2 ab	0.159
		P3	26.82 a	0.033	16.40 ab	0.119	35.0 ab	0.101	572.0 ab	0.130
		P4	26.88 a	0.050	16.40 ab	0.050	35.0 ab	0.103	592.0 ab	0.103
	Si0	P0	22.20 c	0.034	14.40 b	0.056	35.2 ab	0.119	533.6 ab	0.097
		P1	23.70 b	0.025	15.20 ab	0.064	32.4 b	0.074	494.0 b	0.123
		P2	22.44 c	0.023	15.20 ab	0.064	33.0 ab	0.127	499.2 b	0.102
		P3	22.50 c	0.047	15.60 ab	0.096	36.8 ab	0.136	572.8 ab	0.155
		P4	22.32 c	0.043	15.60 ab	0.096	39.6 a	0.183	615.2 a	0.187
ANOVA										
P			ns		ns		ns		ns	
Si			***		ns		ns		ns	
P×Si			ns		ns		ns		ns	
Exp-3	Si1	P0	25.36 c	0.055	18.00 a	0.099	38.40 a	0.078	688.80 a	0.093
		P1	29.41a	0.116	18.00 a	0.070	37.40 a	0.006	675.20 ab	0.115
		P2	26.23 b	0.048	18.00 a	0.050	36.40 a	0.075	655.20 abc	0.075
		P3	25.70 c	0.061	17.60 a	0.045	36.00 a	0.072	632.00 abcd	0.048
		P4	27.38 b	0.016	16.80 b	0.058	36.60 a	0.080	614.40 bcd	0.088
	Si0	P0	26.12 bc	0.097	15.40 d	0.043	37.00 a	0.047	566.00 d	0.045
		P1	27.56 b	0.090	16.20 c	0.025	36.30 a	0.013	581.33 d	0.001
		P2	25.52 c	0.130	16.00 c	0.048	37.67 a	0.013	602.67 cd	0.001
		P3	24.12 d	0.093	17.80 a	0.023	33.00 b	0.023	582.67 d	0.012
		P4	26.24 b	0.037	17.80 a	0.023	33.00 b	0.023	583.33 d	0.039
ANOVA										
P Si P×Si			***		*		*		**	
			**		ns		**		***	
			**		**		ns		ns	

Lowercase letters in the figure indicate significant differences among different treatments determined by Duncan’s test (*P*< 0.05). Two-way analysis of variance (ANOVA) was used to evaluate the effect of P, Si, and their interaction (P×Si) on variables at the significance levels of *P*< 0.05, *P*< 0.01, *P*< 0.001, and no significant difference, represented by “*”, “**”,”***”, and “ns”, respectively. RNPE, row number per ear; KNPR, kernel number per row; GNPE, grain number per ear.

The greatest yield was recorded in the P1Si1 plants in both growth seasons (**Table 1**; *P*< 0.05). The average Cv of yield were lower in Si1 plants than in Si0 plants (0.029 vs. 0.0349; 0.059 vs. 0.089), which indicated that the yield stability was better in Si1 plants than in Si0 plants.

The P and Si fertilizers significantly affected the commercial traits of sweet corn. During the Exp-2 and Exp-3 periods, on average, the Si1 plants had significantly higher ear fresh weight (449 vs. 337; +33.23%), (509.6 vs. 487.6; +4.38%); ear length (31.88 vs. 31.00; +2.83%), (29.64 vs. 28.76; +3.05%); ear diameter (6.37 as. 6.21; +2.58%), (6.75 vs. 6.66; +1.35%); cob fresh weight (328.6 vs. 278.6; +17.94%), (367.84 vs. 365.2; +0.72%); cob length (21.22 vs. 20.37; +4.17%), (20.74 vs. 20.75;+ 0.00%); cob diameter (5.40 vs. 5.32; +1.50%), (5.84 vs. 5.85; + 0.00%) than those in the Si0 plants, respectively (**Figure 12**; *P*< 0.05).

**Figure 12:**
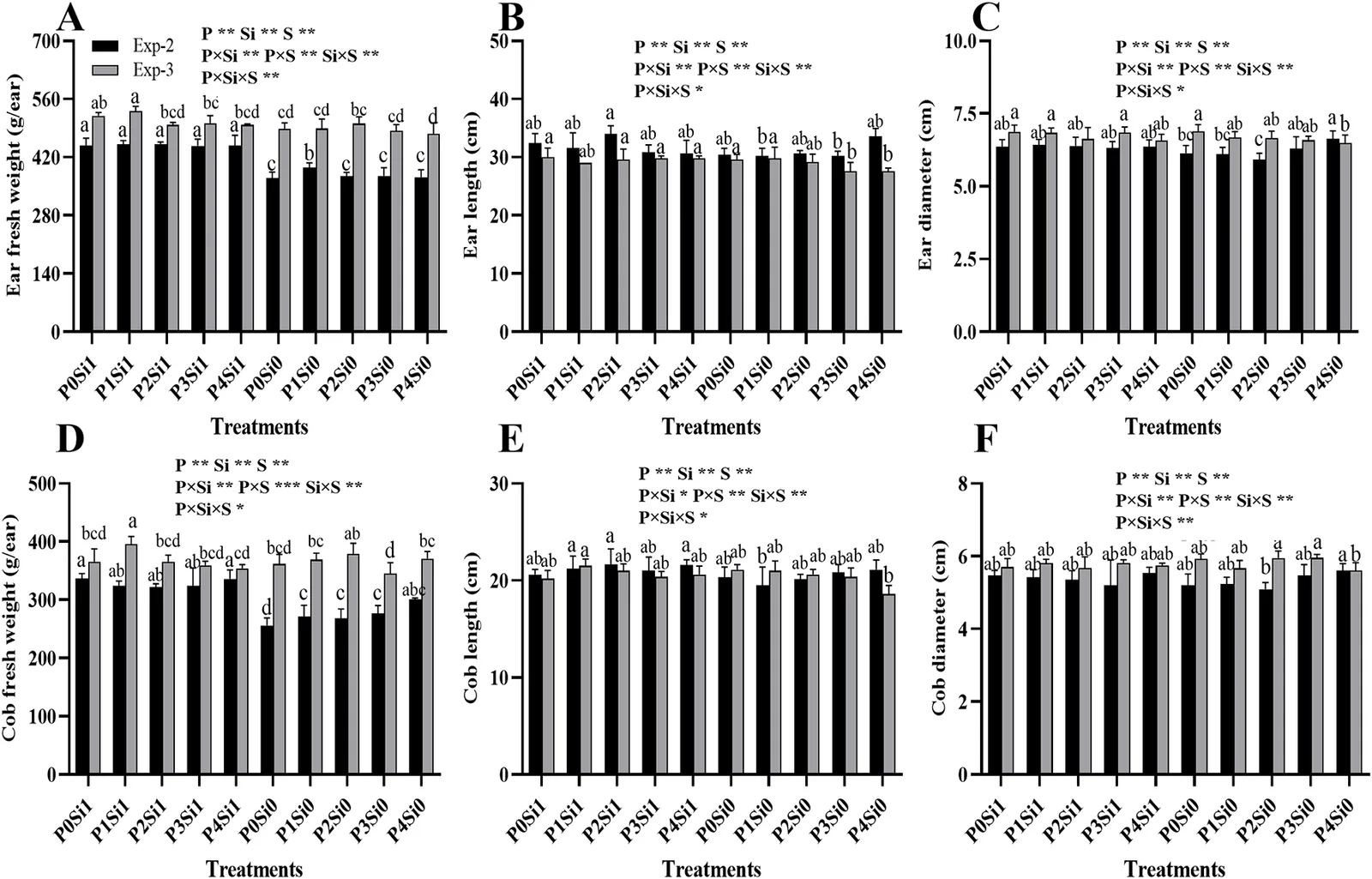
The effect of Si and P fertilizers on the commercial traits of sweet corn ears during the periods corresponding to Exp-2 and Exp-3 is illustrated. Ear fresh weight **(A)**, ear length **(B)**, ear diameter **(C)**, cob fresh weight **(D)**, cob length **(E)**, and cob diameter **(F)**. Vertical bars represent SE values. Lowercase letters indicate significant differences among different treatments determined by Duncan’s test (P< 0.05). Three-way Anova was conducted to evaluate effect of P, Si, Season and their interaction (P×Si×S) on variables at the significance levels of P< 0.05, P< 0.01, P< 0.001, and no significant difference (P > 0.05), represented by “*”, “**”,”***”, and “ns”, respectively.

### 3.9 Pearson correlation analysis and structural equation modeling

Pearson correlation analysis indicated that the BRN (0.37; *P<* 0.001), CRN (0.09; *P > 0.05*), BRWN (0.30; *P<* 0.01), WRA (0.41; *P<* 0.001), NRA (0.34; *P<* 0.001), BRNR (0.29; *P<* 0.01), PH (0.28; *P<* 0.01), third ID (0.22; *P<* 0.05), third ICSA (0.18; *P > 0.05*), BS (0.27; *P<* 0.01), CS (0.40; *P<* 0.001), PS (0.24; *P > 0.05*), and MLAR (0.37; *P<* 0.001) were positively correlated with the ear yield (**Figure 13**). The lodging rate was negatively correlated with BRN (-0.30; *P<* 0.001), BRWN (-0.09; *P > 0.05*), WRA (-0.33; *P<* 0.001), NRA (-0.44; *P<* 0.001), BRNR (-0.63; *P<* 0.001), PH (-0.56; *P<* 0.001), third IL (-0.36; *P<* 0.001), BS (-0.16; *P > 0.05*), CS (-0.23; *P<* 0.05), PS (-0.11; *P > 0.05*), and MLAR (-0.54; *P<* 0.001) (**Figure 13**). Lodging rate was negatively correlated with ear yield (r = -0.46; *P<* 0.001) (**Figure 13A**).

**Figure 13:**
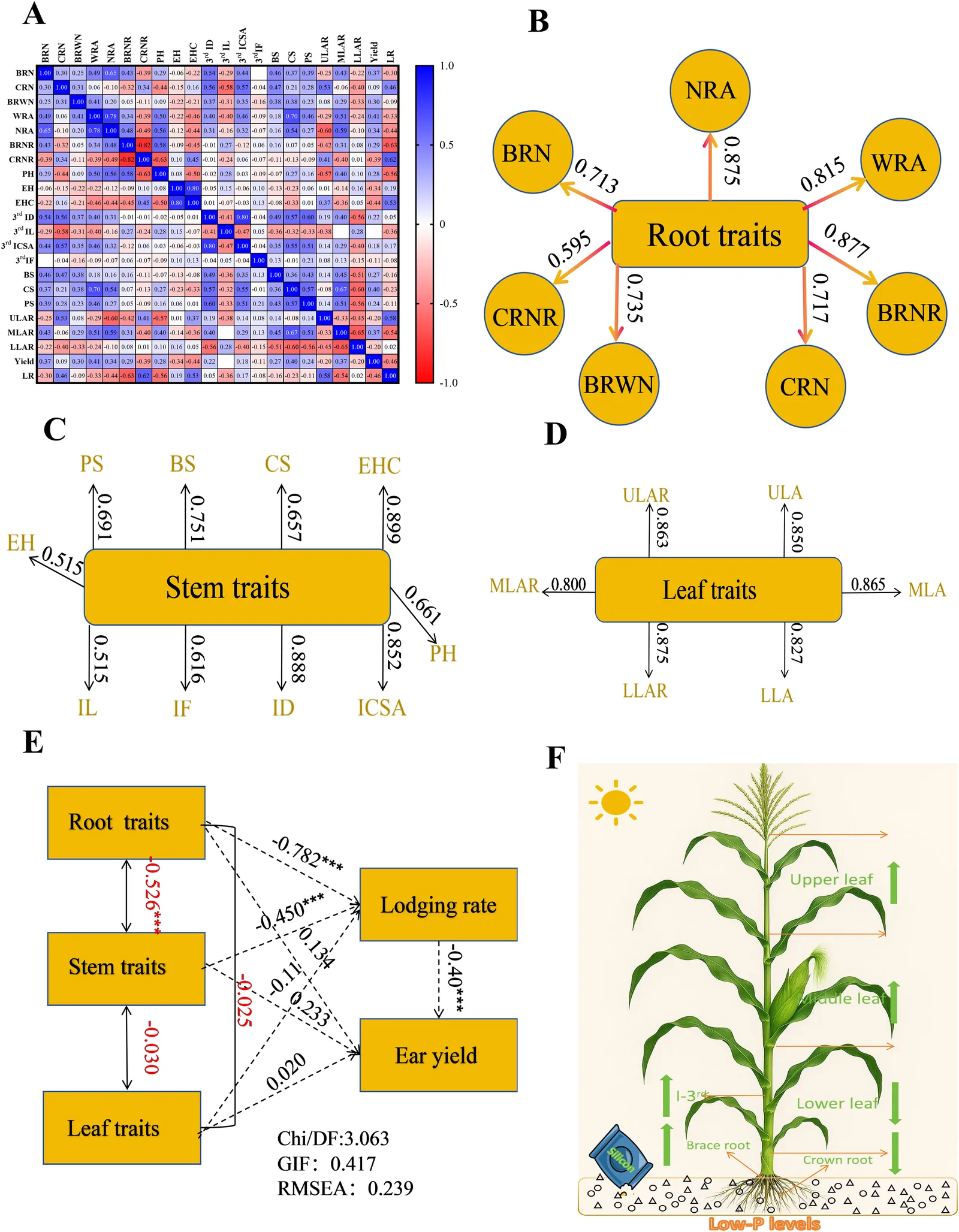
Correlation analysis and structural equation model (SEM) of the relationship among lodging, yield, and the variables. The correlation among root, shoot, leaf development, lodging, and yield **(A)**. The SEM of the relationship among root lodging, yield, and root-shoot-leaf related variables during the three growth seasons **(B–E**), respectively). Mechanism diagram **(F)**. The value in each cell of **(A)** was the correlation coefficient (r); the value in red beside the solid line is the correlations coefficient in the SEM **(E)**, and the standard path coefficient are presented beside the dashed line **(B–D)**; “↑” and “↓” indicate positive and negative effects in the mechanism diagram **(F)**. BRN, brace root number; CRN, crown root number; BRWN, brace root whorl number; WRA, root angle (wide side); NRA, root angle (narrow side); BRNR, brace root number rate; CRNR, crown root number rate; PH, plant height; EH, ear height; EHC, ear height coefficient; 3^rd^ ID, diameter of the third internode; 3^rd^ IL, length of the third internode; 3^rd^ ICAS, cross-sectional area of the third internode; 3^rd^ IF, flatness of the third internode; BS, bending strength; CS, compressive strength; PS, puncture strength; ULA, upper leaf area; MLA, middle leaf area; LLA, lower leaf area; ULAR, upper leaf area ratio; MLAR, middle leaf area ratio; LLAR, lower leaf area ratio; ns, no significance; “*”,”**” and “***” indicate statistical significance at *P<* 0.05, *P*< 0.01, and *P*< 0.001, respectively.

A structural equation model was constructed to elucidate the agronomic mechanism involving the lodging rate, yield, root traits, stem traits, and leaf traits (**Figure 13B-E**). The root traits had the biggest direct effect on the lodging rate (-0.782^***^), with BRNR having the highest loading of 0.877; stem traits (-0.450^***^), with EHC having the highest loading of 0.899; leaf traits (-0.11), with LLAR having the highest loading of 0.875 (**Figure 13B–E**).

The mechanistic model suggests that Si fertilizer enhances both yield and lodging resistance in sweet corn under reduced P supply. This is achieved primarily by promoting brace root differentiation and development, while concurrently suppressing crown root development. Concurrently, improved stem mechanical strength (via third-internode development) and optimized canopy structure (via middle-canopy leaf development) (**Figure 13F**).

## 4 Discussion

### 4.1 Effect of P-Si fertilizer on crop lodging resistance and yield improvement

Sweet corn is an important economic crop in tropical China regions. However, the lodging event occurred frequently throughout all growth seasons due to the severe climatic conditions and unreasonable fertilizer management (Yin et al., 2016). In 1983, Raven suggested that silica could substitute for lignin or cellulose as a structural material in plants, and should be favoured because of its lower energetic costs (Raven, 2003).To date, several studies have shown that silicon (Si) application positively promotes stem lignin biosynthesis, and that increased Si content in stems effectively improves crop lodging resistance and yield. These findings including our present study offer compelling evidence for this view of Raven. P is a crucial nutrient for crop development; most of the applied P can be fixed in farm soil, especially in saline-alkali soil (Ma et al., 2023; Costa et al., 2024). Previous findings have indicated that Si enhances P uptake through two main mechanisms: (i) stimulating the biosynthesis of root exudates, such as malate and citrate, which compete with P for soil adsorption sites; and (ii) forming complexes with aluminum (Al) and iron (Fe), thereby increasing P availability in the soil solution (Costa et al., 2024). In this study, the results indicated that Si fertilizer can improve yield (**Table 1**) and lodging resistance (**Figure 11**) under low-P (P1 in this study; 50 kg/ha P_2_O_5_) input simultaneously, which aligns with the findings in rice plants (Li et al., 2020). Hence we believe that Si-fertilizer might be as a potential limiting factor for phosphorus availability in sweet production in tropical China regions. However, the mechanisms underlying Si-mediated PUE enhancement remain to be further elucidated.

### 4.2 Root differentiation and development on the lodging resistance of sweet corn

The root system architecture (RSA), including root angle, root length, and root diameter, is associated with lodging resistance in crop development (Shao et al., 2018). The root system of cereal crops, including maize, rice, and wheat, is composed of crown roots and brace roots (G. Viana et al., 2022). Crown roots originate from the stem node below ground and mainly play a role in absorption (G. Viana et al., 2022). Brace roots originate from the stem node above the ground and play an important role in anchoring (G. Viana et al., 2022). Hence, balancing the development of these roots is an effective strategy to reduce lodging and consequently achieve high yield-output. Liu et al. (2019) found that an appropriate ratio of crown number and brace root number (CRN: BRN) of 0.66:0.34 is most suitable for maize development, and Liang et al. (2026) also agreed with this finding. BRWN is also an important trait related to root pushing resistance (RPR); Wei et al. (2025) reported that the BRN on whorl 2 contributes more to RPR-intact than that on whorl 1. Our findings indicated that the Si1 plant had significantly larger root angle (**Figure 3**), higher BRN, BRNR (**Figure 2**), and BRWN (**Figure 4**) than the Si0 plants after the same level of P was applied. Among these root traits, the BRNR had the greatest positive effect on lodging resistance, as determined by Pearson correlation analysis and SEM (**Figure 13**). These results aligned with those reported by Liu et al. (2019) and Liang et al. (2026). Moreover, we also found that the structural carbohydrates (lignin, cellulose, and hemicellulose) in brace roots were positively correlated with lodging resistance (**Figure 9**; **Supplementary Figure S4**), which agreed with the findings of other studies (Liu et al., 2021). Current findings suggest that both root system architecture and structural carbohydrate content in roots are the critical factors for improving resistance. Breeders should consider this point when selecting breeding procedures.

### 4.3 Shoot development on lodging resistance and yield

Lodging resistance is closely associated with several plant morphological traits, including plant height, CGH, basal internode length, stem diameter, and culm wall thickness (Niu et al., 2021). Zhang et al. (2022) demonstrated that lodging-resistant maize prioritizes photosynthate allocation to the basal stem and roots, fueling the biosynthesis of structural carbohydrates. This process concurrently enhances stem mechanical strength and root-soil anchorage. Hence, leaf senescence should be delayed to coordinate the ear (sink) development and lodging resistance during the filling stage (Ren et al., 2024). Leaf angle and leaf area distribution throughout the plant canopy are the most important factors for determining plant architecture. Many studies have investigated the aspect of leaf angle to modulate canopy structure and popularized the term “smart canopy architecture”, where plants with a compact upper canopy and spreading lower canopy can improve lodging resistance and yield by coordinating the light distribution and maximizing photosynthetic capacity (Zhou et al., 2025). However, the effect of leaf area distribution through the canopy layers on crop lodging resistance and yield, conversely, captured little attention. In this study, plants in the Si1 plots exhibited shorter stature, lower ear height, reduced ear height coefficient (EHC), and thicker but shorter stem nodes compared to those in the Si0 plots (**Figures 5, 6**). Additionally, Si1 plants showed a greater proportion of leaf area in the middle canopy (**Figure 8**), higher structural carbohydrate content in stem nodes (**Figure 9**), and increased SPAD and nitrogen content in the ear leaf (**Supplementary Figure S5**). Collectively, these traits are consistent with the enhanced lodging resistance and yield performance previously documented (Niu et al., 2021). Leaf angle was also measured across canopy layers (**Supplementary Figure S6**). Relative to Si0, Si1 plants under low-P conditions exhibited a smaller upper-canopy leaf angle but a larger middle-canopy leaf angle, with no significant difference observed in the lower canopy. Correlation analysis further revealed that plant height (r =-0.56) and the proportion of leaf area in the middle canopy (r =-0.54) showed the strongest negative associations with lodging resistance (**Figure 13**).

These findings highlight that coordinating stem and leaf development is essential for simultaneously improving lodging resistance and yield in sweet corn.

Beyond stem traits, leaf angle and its vertical distribution within the canopy emerged as equally critical determinants of an optimized canopy architecture. Specifically, a “smart canopy” characterized by smaller upper-leaf angles, larger middle-leaf angles, and an increased proportion of leaf area allocated to the middle layer may confer dual benefits: it reduces wind-induced disturbance and lowers the center of gravity height to enhance lodging resistance, and supports both ear sink development and basal stem maintenance.

### 4.4 Relationship among soil activity, root differentiation, lodging resistance, and yield

Soil activity is closely associated with root architecture and the absorption of nutrients and water (Li et al., 2016). Among the parameters of soil activity, the SUR, SSU, and SPH are strongly correlated with nitrogen metabolism, sucrose metabolism, and phosphorus metabolism, respectively (**Figure 10**). The root architecture, such as root length, root surface area, and root tip number, is positively correlated with soil activity (Gao et al., 2025). However, the relationship between soil activity and root differentiation, lodging, and yield was unclear. In this study, we found that the Si fertilizer applied with low-P (P0 and P1) significantly increased the activity of SUR, SSU, SPH, and soil POD, which was positively correlated with BRN and BRNR (**Supplementary Figure S7**). These findings indicated that Si fertilizers with low-P management can trigger brace root formation by improving N, P, and sucrose metabolism, thereby maximizing the lodging resistance and yield of sweet corn (**Supplementary Figure S7**). However, the mechanism underlying root differentiation needs to be further studied because the soil-rhizosphere is a complex system that is modulated by root exudates and rhizosphere microorganisms; thus, their roles in sweet corn root system differentiation should be further investigated.

### 4.5 Yield, yield component, and commercial traits

The 2025 growing season (Exp-1) was severely disrupted by Super Typhoon Kajiki (wind speed: 50 m s^-^¹) on August 24 during the filling stage, resulting in complete crop damage and preventing harvest. Grain yield in sweet corn is primarily determined by the number of kernel rows per ear (RNPE) and the number of kernels per row (KNPR). RNPE is established during spike formation at the jointing stage, whereas KNPR largely depends on the post-anthesis grain-setting rate (Chang et al., 2026). Under the same P fertilizer regime, plants in Si1 plots exhibited significantly higher yield than those in Si0 plots (**Table 1**), driven by simultaneous increases in both RNPE and KNPR (i.e., total grain number). Notably, the coefficient of variation (Cv) for these yield components was lower in Si1 than in Si0 plants (**Table 1**), suggesting that Si application promotes kernel formation while stabilizing population-level yield—a finding consistent with previous reports (Dong et al., 2024). Beyond yield quantity, ear appearance traits—which affect commercial quality and harvest index—were also improved by Si application. Si1 plants had significantly greater ear fresh weight, ear length, ear diameter, cob fresh weight, cob length, and cob diameter than Si0 plants (**Figure 11**), indicating that Si facilitates desirable ear and cob (de-husked ear) development.To dissect the relative contributions of different organ groups to yield, structural equation modeling (SEM) was employed. The results revealed that stem traits exerted the largest direct positive effect on yield (standardized path coefficient = 0.223), followed by root traits (0.134) and leaf traits (0.020). In contrast, lodging rate showed a strong negative direct effect on yield (standardized coefficient = –0.400). Collectively, these SEM results indicate that shoot development is the primary determinant of yield, while coordinated root–shoot development is essential for optimizing both lodging resistance and yield, corroborating the findings of Zhang et al. (2022) and Liang et al. (2026).

### 4.6 Perspectives and limitation

Global climate change, and soil erosion are intensifying the frequency of lodging, thereby threatening food security and agricultural production (Thuy and Niem, 2025; Makumi et al., 2025; Hasddin and Ulyasniati, 2025). Based on the findings of this study, we propose that Si fertilization is a beneficial strategy for improving both lodging resistance and yield in sweet corn under low-P conditions. This beneficial effect is attributed to the role of Si in coordinating root–shoot development: on the one hand, it balances root system differentiation and development, particularly for brace roots; on the other hand, it enhances stem node development and optimizes canopy architecture. However, the regulatory mechanisms underlying brace root formation and development—such as the involvement of endogenous hormone networks—as well as the mechanisms of Si-enhanced phosphorus use efficiency, require further investigation.

## 5 Conclusion

In this study, we demonstrated that Si fertilization at low-P levels (P1: 50 kg ha^-^¹ P_2_O_5_) simultaneously enhances lodging resistance and yield in sweet corn. These findings indicated that: (i) root lodging was the main threat to sweet corn production in tropical China, and Si fertilizer can improve root lodging resistance by promoting brace root development—exerted the largest positive direct effect on lodging resistance; (ii) plant height and middle-canopy leaf area distribution increased the yield, the lodging risk was offset by enhanced stem development under Si fertilizer. Our study provide reasonable Si fertilizer strategy to sweet corn production under low P condition, and as well as a phenotypic framework for breeding programs targeting improved standability and productivity in tropical sweet corn production systems. However, given that our experiments were conducted at a single location, multi-site validation across representative tropical regions of China (e.g., Guangdong, Yunnan, Guangxi, and Taiwan) is warranted to assess the environmental robustness of the observed Si effects. Nevertheless, our results establish a scientific basis for integrating Si fertilization into sweet corn management practices in tropical China.

## Data Availability

The original contributions presented in the study are included in the article/**Supplementary Material**. Further inquiries can be directed to the corresponding author.
