# Supplementary material for: Simultaneous enhancement of sweet corn yield and lodging resistance via silicon application with low-phosphorus management under dense planting in tropical China
Source: Front Plant Sci. 2026 Oct 20;17:1919054. doi: 10.3389/fpls.2026.1919054 (PMC13727456; doi:10.3389/fpls.2026.1919054)
Supplement: Supplementary file 1 [file SupplementaryFile1.doc]

Fig. S1 The appearance and source of Si fertilizer.

Fig. S2 Simulate the wind disturbance (1.5 m over the tassel; 15 m/s wind speed ) by DJI drones to evaluate sweet corn lodging resistance.

Fig. S3 lodged plants that cause by super typhoon “Kajiki” (photographed at Yazhou district, Sanya City; 25 Aug, 2025 ).

Fig. S4 Person analysis between structure carbohydrates and lodging rate. SLC, stem lignin content; RLC, root lignin content; SCC, stem cellulose; RCC, root cellulose; SHCC, stem hemi-cellulose; RHCC, root hemi-cellulose, LR, lodging rate. Value in each cell was the correlation coefficient (r), the red and blue cells indicated negatively correlation and positive correlation, respectively.

Fig. S5 Effect of Si and P fertilizer on SPAD and ear leaf nitrogen content in sweet corn during Exp-2 and Exp-3. SPAD (A), Nitrogen content in ear leaf in sweet corn (B). Vertical bars represent SE values. Lowercase letters in the figure indicate significant differences among different treatments determined by Duncan's test (*P* < 0.05). Two-way analysis of variance (ANOVA) was used to evaluate the effect of P, Si and their interaction (P×Si) on variables at the significance levels of *P* < 0.05, *P* < 0.01, *P* < 0.001 and no significant difference, represented by “*”, “**”,“***” and “ns”, respectively.

Fig. S6 Effect of Si and P fertilizer on leaf angle through canopy layers of sweet corn (average over the three growth seasons). Upper leaf angle (A), Middle leaf angle (B), lower leaf angle (C). Vertical bars represent SE values. Lowercase letters in the figure indicate significant differences among different treatments determined by Duncan's test (*P* < 0.05). Two-way analysis of variance (ANOVA) was used to evaluate the effect of P, Si and their interaction (P×Si) on variables at the significance levels of *P* < 0.05, *P* < 0.01, *P* < 0.001 and no significant difference, represented by “*”, “**”,“***” and “ns”, respectively.

Fig. S7 Person analysis between soil enzymes activities and root development. BRN, brace root number; CRN, crown root number; BRNR, brace root number ratio; CRNR, crown root number ratio; WRA, root angle (wide side); NRA, root angle (narrow side); SUR, soil urease activity; SSU, soil sucrase activity; SPH, soil phosphatase activity; SCL, soil cellulase activity; POD, soil peroxidase; LR, lodging rate. Value in each cell was the correlation coefficient (r), the red and blue cells indicated negatively correlation and positive correlation, respectively.
